# Nuclear Factor Y controls nutrient-adaptive epithelial growth by regulating mTOR in the *Drosophila* midgut

**DOI:** 10.1242/dev.205643

**Published:** 2026-07-13

**Authors:** Tetiana Strutynska, Onur Deniz, Jaakko Mattila

**Affiliations:** ^1^ Faculty of Biological and Environmental Sciences, University of Helsinki, Helsinki 00790, Finland; ^2^ Institute of Biotechnology, University of Helsinki, Helsinki 00790, Finland

**Keywords:** *Drosophila* midgut, Tissue growth, NF-Y, mTOR, Intestinal stem cell

## Abstract

The intestinal epithelial lining is highly dynamic, with size and cellular composition adapting to nutrient status. This requires regulation of intestinal stem cell (ISC) proliferation and enterocyte size. How the intestinal absorptive area matches physiological nutrient conditions remains unclear. Here, we show that the transcription factor Nuclear Factor Y (NF-Y) plays a role in this process. NF-Y loss of function in ISCs led to high proliferation and cell growth, a phenotype influenced by dietary nutrients. NF-Y loss of function also increased nutrient metabolism, as shown by more mitochondria and larger lipid droplets in progenitors. Mechanistically, NF-Y restrains mTOR complex 1 (mTORC1) activity in ISCs by controlling transcription of mTORC1 signaling components such as Pras40 and Sestrin. Overall, our results demonstrate that NF-Y limits excessive nutrient-adaptive intestinal epithelial growth.

## Teaser

**Summary:** Loss-of-function studies reveal a molecular mechanism that limits intestinal growth in response to nutrients, helping maintain tissue balance and prevent harmful overgrowth.

## INTRODUCTION

The size of the intestinal epithelial lining is dynamically regulated to match the digestive and metabolic needs of an animal. For example, the *Drosophila* midgut, the counterpart of the mammalian small intestine, is highly adaptive to nutritional changes. When calorie-restricted, intestinal stem cell (ISC) proliferation is reduced, and the midgut absorptive area decreases due to enterocyte (EC) loss and reduced cell size. Upon feeding, the proliferation and differentiation of ISCs increase, and the ECs enlarge, leading to an increase in absorptive epithelial area (Bonfini et al., 2021; Mattila et al., 2024; O'Brien et al., 2011). Although the cellular processes of midgut nutrient adaptation have been described in fine detail, the mechanisms underlying the regulation of optimal epithelial size under physiological conditions remain poorly understood.

Adaptive regulation of intestinal absorptive area is mediated by nutrient-sensing pathways, including the amino acid-sensing mTOR complex 1 (mTORC1) signaling pathway (Bonfini et al., 2021; Mattila et al., 2024; O'Brien et al., 2011). In ISCs, mTORC1 regulates anabolic metabolism, enabling cell growth and division, while in progenitors, mTORC1 activity is linked to lineage choice and differentiation (Haller et al., 2017; Kapuria et al., 2012; Mattila et al., 2024; Sampson et al., 2016). In niche cells, mTORC1 functions cell non-autonomously to regulate the proliferative activity of stem cells (Yilmaz et al., 2012). Thus, mTORC1 has a profound and complex role in adaptive regulation of the intestinal epithelium. Unrestricted mTORC1 activation can have catastrophic consequences for cells (Liu and Sabatini, 2020). Therefore, feedback mechanisms that limit mTORC1 activity have evolved. These mechanisms ensure optimal mTORC1 activity, balancing cellular growth and division to available nutrients, growth factors and energy (Goul et al., 2023). However, how mTORC1 activity is balanced to achieve optimal epithelial size in physiological conditions, especially in complex tissues such as the *Drosophila* midgut, remains enigmatic.

Nuclear Factor Y (NF-Y) is a conserved and pleiotropic trimeric transcription factor composed of the subunits NF-YA, NF-YB and NF-YC (Li et al., 2018). NF-Y regulates gene expression by binding to the CCAAT box via NF-YA, the DNA-binding subunit of the complex (Nardini et al., 2013). NF-Y plays an essential role in early zygotic development in mammals and is a key driver of cell-cycle progression in proliferating cells, including hematopoietic and muscle stem cells (Benatti et al., 2011; Bungartz et al., 2012; Hu et al., 2006; Liu et al., 2019; Rigillo et al., 2021). Despite these established functions in development and the cell cycle, little is known about the physiological roles of NF-Y transcription factors. Here, we report an unexpected role for NF-Y in regulating *Drosophila* midgut epithelial growth. Specifically, we show that NF-Y inhibits ISC proliferation, mitochondrial biogenesis and lipid accumulation in progenitors, thereby protecting the intestinal epithelial lining from excessive growth and epithelial disruption. Mechanistically, NF-Y limits mTORC1-mediated ISC activation by regulating transcription of mTORC1 signaling components, such as Pras40 and Sestrin. Through this mechanism, we identify NF-Y as a key regulator of intestinal epithelial cells under conditions of high growth demand.

## RESULTS

### Nuclear Factor Y restricts ISC activity cell autonomously

**Fig. 1.**
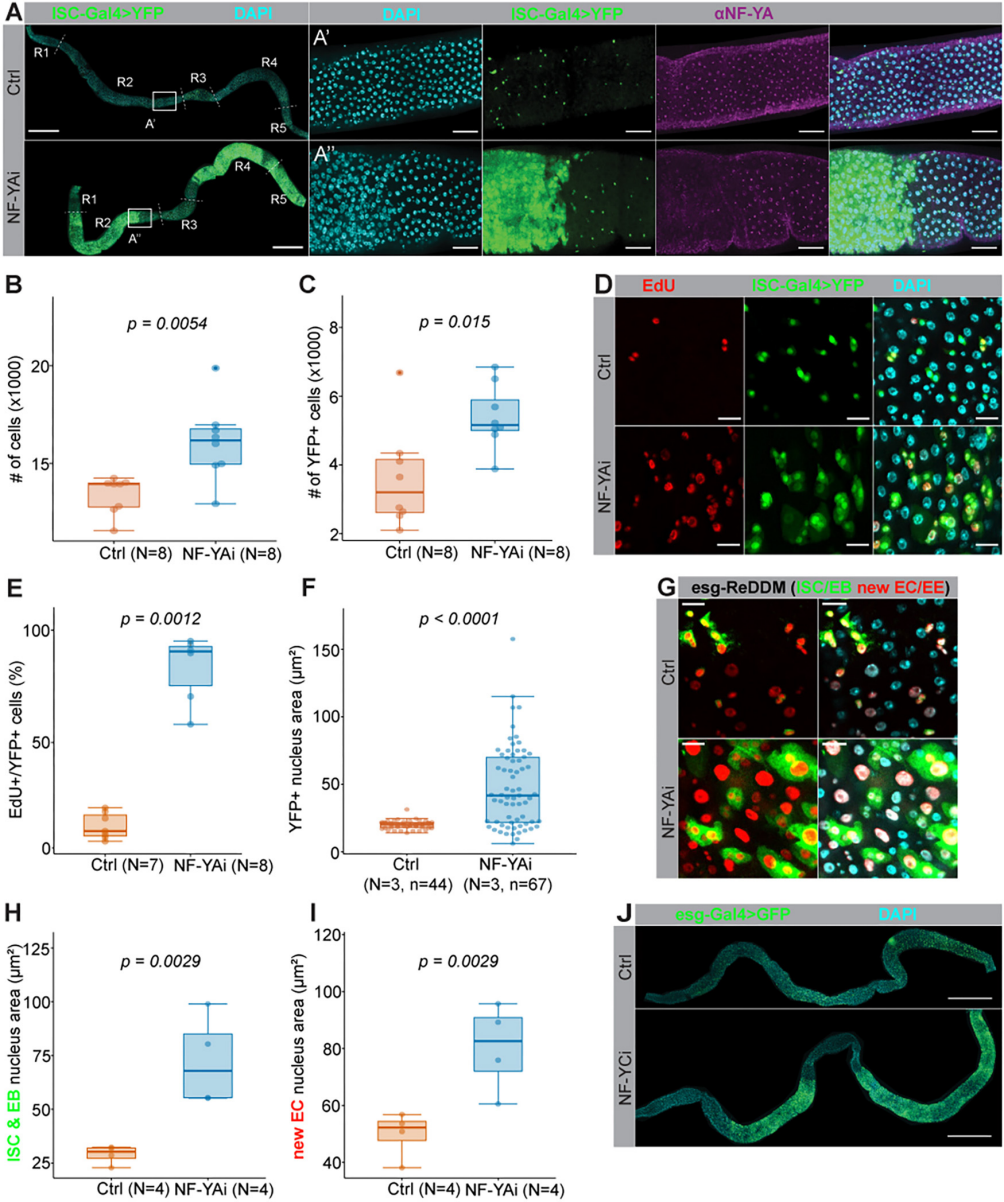
**NF-Y restricts intestinal stem cell proliferation and progenitor growth.** (A) Representative images of esg-Gal4^ts^, Su(H)GBE-Gal80>NF-YA RNAi (KK) and control female midguts. Scale bars: 500 μm. (A′,A″) The R3-R4 border regions outlined in A stained by using anti-NF-YA antibodies (magenta). Scale bars: 50 μm. Flies were kept at +18°C for 3 days in minimal 2% sucrose media, shifted to a permissive temperature (+29°C) for 3 days to induce RNAi, and then kept on a holidic diet for an additional 7 days. (B,C) Quantification of midgut total cell numbers (B) and YFP-positive cells (C) from the experiment depicted in A. (D) Representative images of esg-Gal4^ts^, Su(H)GBE-Gal80>NF-YA RNAi and control female midguts stained with EdU. Scale bars: 20 μm. Images are of the R4b region after 3 days on a holidic diet followed by a 16 h pulse on a holidic diet supplemented with EdU. (E,F) Quantification of the number of EdU-positive intestinal stem cells (ISCs) (E) and the nuclear area of YFP-positive cells (F) from the experiment depicted in D. (G) Representative images of the esg-Reddm NF-YA RNAi and control female flies from the R4b region after tracing for 7 days on a holidic diet. Scale bars: 20 μm. ISCs and enteroblasts (EBs) are in green; newly formed enterocytes (ECs) and enteroendocrine (EE) cells (small nuclei) are in red. (H,I) Quantification of nuclear area of ISCs and EBs (H), and newly formed ECs (large nuclei RFP^+^ cells) (I) from the experiment depicted in G. (J) Representative images of esg-GAL4^ts^>NF-YC RNAi and control female midguts. Scale bars: 500 μm. *P*-values were obtained using the Wilcoxon rank-sum test. Box plots show the median (line), interquartile range (box) and Tukey-style whiskers.

By systematically analyzing transcriptional regulators, we discovered a previously unreported role for Nuclear Factor Y subunit Alpha (NF-YA) in the adult *Drosophila* midgut. When knocked down by RNA interference (RNAi) in female flies using the ISC-specific driver esg-Gal4ts, Su(H)-Gal80 coupled with UAS-YFP (hereafter referred to as ISC-Gal4), we observed a substantial expansion of the YFP-expressing cell pool compared with control animals (Fig. 1A). The YFP-positive cells accumulate in a highly region-specific manner in regions R2, R4 and R5, whereas regions R1, R3 and the flanking border regions are unaffected (Fig. 1A). These data was further confirmed using an independent RNAi line, which produced a similar region-specific expansion of the YFP-positive cells (Fig. S1A). Quantification of the phenotype showed significantly increased total and YFP-positive cell numbers (Fig. 1B,C). Staining with NF-YA antibodies confirmed the loss of NF-YA protein from cells expressing the RNAi reagent (Fig. 1A). The increase in cell numbers suggests an elevated rate of ISC proliferation in response to NF-YA loss of function. To test this possibility, we used 5-ethynyl-2'-deoxyuridine (EdU) to label cells with newly synthesized DNA. In control animals, a 16 h EdU pulse labels YFP-negative enteroblasts (EBs) and small YFP-positive ISCs that have begun endoreduplication or S-phase, respectively (Fig. S1B). NF-YA knockdown by the ISC-Gal4 driver resulted in a significant increase in the number of EdU-positive ISCs compared with controls, indicating increased proliferative activity (Fig. 1D,E).

NF-YA localizes to the nucleus of ISCs, as well as in the polyploid enterocytes (ECs) (Fig. 1A and Fig. S1C). Thus, we asked whether NF-YA regulates ISCs cell-autonomously or through non-cell-autonomous effects from the EB/EC population. To this end, we used the Su(H)-Gal4 and Myo1A-Gal4 drivers to knock down NF-YA in the EBs and ECs, respectively. Knocking down NF-YA in the EB/EC population did not lead to noticeable expansion of the progenitor cell pool (Fig. S1D,E). Interestingly, NF-YA knockdown in ISCs led to a broad size distribution of YFP-positive cells, compared with the uniformly small cell sizes in control animals (Fig. 1F). As a surrogate for cell size, we measured the maximum nuclear cross-sectional area, which we have previously shown to correlate with cell size in the adult midgut (Mattila et al., 2024). In control animals, ISC-Gal4-driven YFP expression is restricted to diploid ISCs by the EB-specific expression of Gal80 (Su(H)-Gal80). However, following NF-YA loss, many YFP-positive cells enlarge, suggesting rapid growth and retention of the YFP signal. To determine whether these cells are progenitors or mature progeny, we first stained midguts with antibodies against HRP, a known progenitor marker of the *Drosophila* midgut (O'Brien et al., 2011). Interestingly, many of the enlarged YFP-positive cells are also anti-HRP-positive, suggesting that, despite their large size, some of the NF-YA LOF cells remain as undifferentiated progenitors (Fig. S1F). To further explore the effect of NF-YA loss on ISC differentiation, we used antibodies against Prospero or Pdm1 to identify mature enteroendocrine (EE) cells or ECs, respectively. We observed many YFP-positive cells expressing Prospero or Pdm1 markers, suggesting that NF-YA is not required in ISCs for differentiation (Fig. S1G,H).

To ask whether the enlarged progenitors also produce larger progeny, we employed the esg-ReDDM (repressible dual differential stability cell marker) lineage-tracing method to distinguish between esg-expressing progenitors and fully matured progeny (Antonello et al., 2015). The esg-ReDDM utilizes the differing stabilities of the short-lived membrane-targeted GFP, which acts as a precise temporal marker of esg-Gal4 activity, and the long-lived histone 2B-tagged RFP, enabling tracking of newly differentiated progeny. Our analysis shows that NF-YA knockdown by the esg-gal4 driver results in a significant increase in progenitor and progeny size compared with controls (Fig. 1G-I). Thus, based on our findings, we conclude that NF-YA functions in ISCs to inhibit progenitor growth and progeny size.

NF-YA is the sequence-binding motif of the conserved heterotrimeric Nuclear Factor Y (NF-Y) transcription factor, which forms a functional trimer with the NF-YB and NF-YC subunits (Li et al., 2018). To confirm that the NF-YA LOF phenotype in ISCs results from the disruption of the NF-Y complex, we knocked down NF-YC using esg-Gal4-driven RNAi. Indeed, knockdowns of NF-YA and NF-YC produced identical, region-specific phenotypes, confirming that disruption of the functional NF-Y trimer underlies these phenotypes (Fig. 1J). In conclusion, our results show that NF-Y has a cell-autonomous role in restricting ISC proliferation and progenitor growth in a region-specific manner.

### NF-Y regulates epithelial growth in a nutrient-dependent manner

**Fig. 2.**
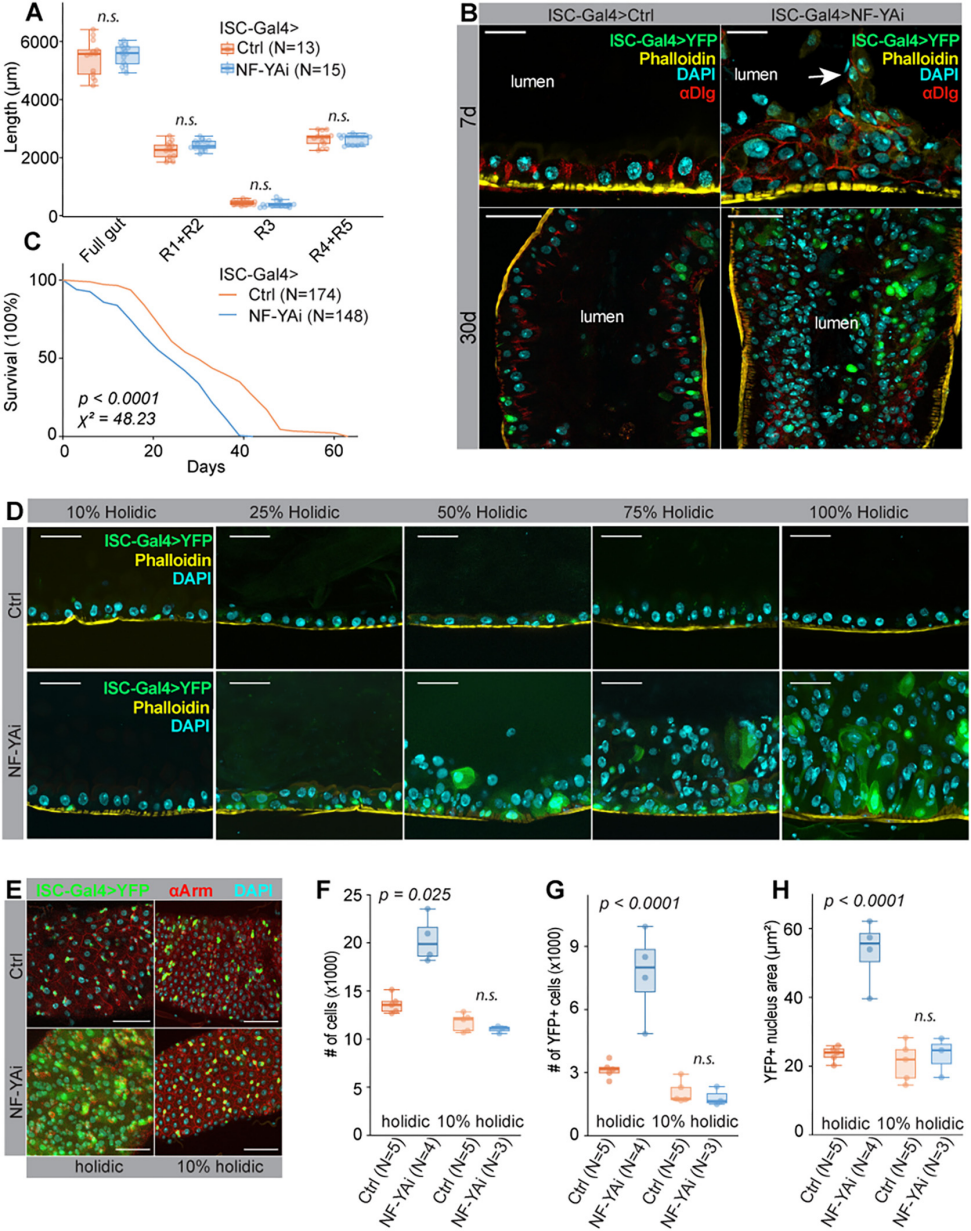
**NF-Y regulates nutrient-adaptive epithelial growth.** (A) Quantification of esg-Gal4^ts^, Su(H)GBE-Gal80>NF-YA RNAi (KK) and control midgut region lengths. (B) Representative images of esg-Gal4^ts^, Su(H)GBE-Gal80>NF-YA RNAi (KK) and control midgut cross-sections from the R4b region immunostained using anti-Dlg antibodies (septate junctions) and phalloidin (F-actin). Flies were kept for 7 days (young) or 30 days (old) on a holidic diet at +29°C. Scale bars: 20 μm (young); 50 μm (old). An arrow indicates a group of cells delaminating from the epithelium. (C) Quantification of esg-Gal4^ts^, Su(H)GBE-Gal80>NF-YA RNAi (KK) and control virgin female survival at +29°C. (D) Representative R4b midgut cross-sections stained with phalloidin from esg-Gal4^ts^, Su(H)GBE-Gal80>NF-YA RNAi (KK) and from control flies fed holidic food at concentrations of 10%, 25%, 50%, 75% and 100%. Scale bars: 20 μm. (E) Representative images of esg-Gal4^ts^, Su(H)GBE-Gal80>NF-YA RNAi (KK) and control midguts from the R4b region from flies kept either on a full holidic diet or on a 10% holidic diet and immunostained using anti-Armadillo antibodies. Scale bars: 50 μm. (F-H) Quantification of total cell numbers (F), YFP^+^ cell numbers (G) and YFP^+^ nuclear area (H) from the experiment depicted in E. Results in A were tested for significance using a two-way ANOVA followed by Tukey's test. *P*-value and chi-squared value in C were obtained using the Log-rank (Mantel-Cox) test. The *P*-value in F was obtained by using a Welch ANOVA followed by the Games-Howell test. *P*-values in G and H were obtained using a two-way ANOVA followed by Tukey's test. Box plots show median (line), interquartile range (box) and Tukey-style whiskers.

The increase in midgut cell number and cell size upon NF-Y disruption in ISCs suggests an expansion of tissue size. However, measuring midgut region lengths in animals with ISC-Gal4-driven NF-YA RNAi revealed no significant differences compared with controls (Fig. 2A). We then asked how the midgut epithelial structure is altered by the increase in cell number and cell size in these animals. Interestingly, the apico-basal epithelial structure in the midguts of NF-YA knockdown animals showed dramatic cellular multilayering when compared with the single-layer organization in control midguts (Fig. 2B). In addition, knockdown of NF-YA resulted in cellular delamination from the epithelium into the lumen (Fig. 2B). Thus, the excess oversized cells in the ISC-Gal4-driven NF-YA knockdown are multilayering and delaminating from the epithelium. The phenotype became more severe with age, resulting in a strikingly narrowed gut lumen (Fig. 2B). To test the physiological relevance of the distorted epithelial structure, we monitored the lifespan of flies with NF-YA knockdown driven by the ISC-Gal4 driver. Indeed, these animals showed a significantly shorter lifespan than controls (Fig. 2C). Thus, NF-Y is important for maintaining optimal epithelial size, and its disruption can reduce animal survival. While searching for physiological conditions that modulate the NF-YA LOF phenotype, we found that epithelial thickening was strongest at undiluted holidic diet (nutrient rich; Piper et al., 2014) and diminished with lower dietary nutrient concentrations (Fig. 2D). Notably, at 10% holidic diet (nutrient poor), the epithelial thickening, increase in proliferation and cell growth upon NF-YA loss of function were completely abolished (Fig. 2D-H), indicating that nutrients are necessary for the NF-YA loss-of-function phenotype. Because the NF-YA loss-of-function phenotype depended on nutrient availability, we next tested whether NF-Y is nutrient regulated. Using ectopic NF-YA with a C-terminal GFP tag as a reporter (Kudron et al., 2018), we found that NF-YA protein levels were upregulated under fed conditions in small-nucleus cells compared with starved controls (Fig. S2A,B). This upregulation supports the causal link between nutrient availability, NF-YA expression and nutrient-induced growth. Together, our results show that NF-Y inhibits ISC activation and epithelial growth in response to increased nutrient intake.

### NF-Y regulates mitochondrial biogenesis and lipid storage in ISCs

**Fig. 3.**
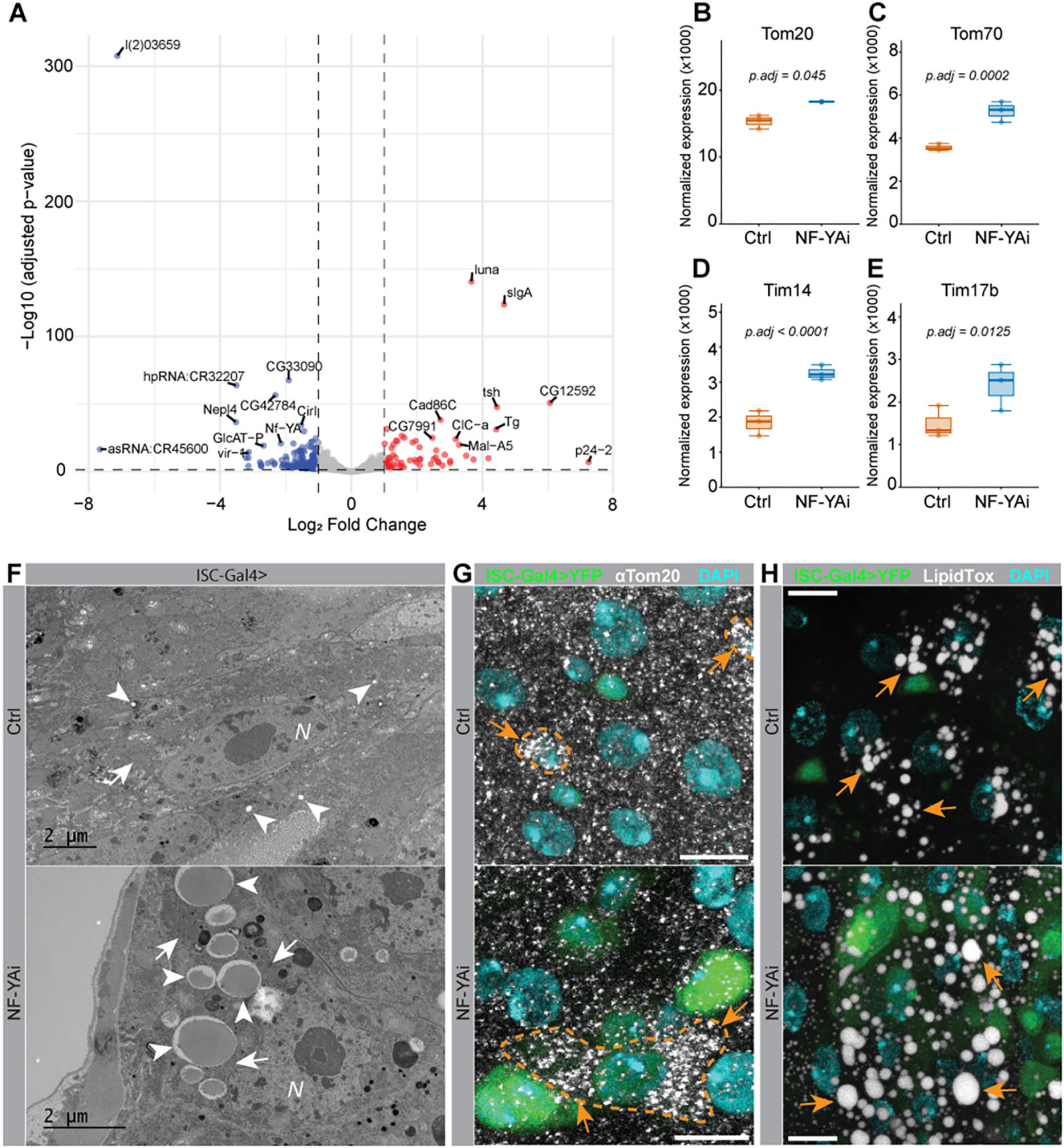
**NF-Y regulates mitochondrial biogenesis and lipid storage.** (A) Volcano plot representing differentially expressed genes between esg-Gal4^ts^, Su(H)GBE-Gal80>NF-YA RNAi (KK) and control intestinal stem cells (ISCs). Significant changes (*P*<0.05 and log2 fold changes>±1) are shown in red (upregulated in NF-YA RNAi) and blue (downregulated in NF-YA RNAi). The 10 most up- and downregulated genes are annotated on the plot. (B-E) Normalized mRNA expressions of Tom20 (B), Tom70 (C), Tim14 (D) and Tim17b (E). (F) Electron micrograph from 30-day-old esg-Gal4^ts^, Su(H)GBE-Gal80>NF-YA RNAi (KK) and control midguts from the R5 region. Arrows indicate lipid droplets; arrowheads indicate mitochondria. (G) Representative images of 7-day-old esg-Gal4^ts^, Su(H)GBE-Gal80>NF-YA RNAi (KK) and control midguts from the R4b region immunostained with anti-Tom20 antibodies. Arrows and outlines indicate Tom20-positive puncta. Scale bars: 10 μm. (H) Representative images of 7-day-old esg-Gal4^ts^, Su(H)GBE-Gal80>NF-YA RNAi (KK) and control midguts from the R4b stained with LipidTox. Arrows indicate lipid droplets. Scale bars: 10 μm. Adjusted *P*-values in A-E were obtained by the DESeq2 package with Benjamini-Hochberg correction. Box plots show median (line), interquartile range (box) and Tukey-style whiskers.

To obtain a global view of the biological processes regulated by NF-Y, we performed mRNA sequencing in NF-YA-deficient ISCs. To restrict our analysis to early regulatory events, we isolated mRNA from ISCs after 3 days of RNA interference induction. At this time, the NF-YA knockdown phenotype is not yet fully penetrant, suggesting that the observed changes in gene expression are directly regulated by NF-Y (Fig. S3A). To isolate ISCs, we used fluorescence-activated cell sorting of YFP-marked ISCs. From the dataset, we performed differential gene expression (DEG) analysis and identified 850 upregulated and 989 downregulated genes, including NF-YA among the ten most downregulated genes (Fig. 3A and Table S1). Gene set enrichment analysis (GSEA) revealed that the most affected biological processes are mitochondrial translation and transcription, and the cell cycle (Fig. S3B). Indeed, the expression of several key cell cycle drivers, such as cyclin B, cyclin E, cdk1 and PCNA, was significantly upregulated upon NF-YA loss of function in ISCs (Fig. S3C-F). The elevated transcription of those genes is consistent with the hyperproliferation of ISCs resulting from NF-Y disruption. In addition, the expression of several components of the inner (Tim proteins) and outer (Tom proteins) membrane translocase complexes of the mitochondrion was significantly upregulated in NF-YA loss-of-function ISCs, including Tom20, Tom70, Tim14 and Tim17b (Fig. 3B-E). These data suggest an increased number or volume of mitochondria in NF-YA loss-of-function ISCs. In line with this, several genes involved in mitochondrial metabolism, including those in the TCA cycle and oxidative phosphorylation, were also significantly upregulated by NF-Y disruption (Fig. S3G). Finally, the expression of genes promoting lipid droplet formation, such as *seipin* and *schlank*, as well as the transcription factor *sugarbabe*, which regulates genes involved in *de novo* lipogenesis (Mattila et al., 2015), was upregulated upon NF-YA loss of function in ISCs (Fig. S3H-J). In conclusion, NF-Y regulates the expression of genes involved in the cell cycle, mitochondrial metabolism, and lipid synthesis and storage in ISCs.

To directly assess the role of NF-Y role in regulating cellular processes, we examined subcellular structures by transmission electron microscopy (TEM). We analyzed midguts from ISC-Gal4-driven NF-YA RNAi animals at 30 days of age, ensuring that the epithelium had been fully turned over by progenitors derived from NF-Y loss-of-function ISCs. In line with the observed gene expression changes, we observed increased mitochondrial number and size upon NF-Y disruption compared with controls (Fig. 3F). Strikingly, the mitochondria surrounded greatly enlarged lipid droplets (Fig. 3F). To exclude the possibility that the observed cellular changes were specific to old age, we immunostained midguts from 7-day-old animals using antibodies against the mitochondrial import receptor subunit Tom20 and the neutral lipid stain LipidTox. Consistent with the TEM analysis, we observed an increased number of anti-Tom20 immunostained puncta and larger lipid droplets in ISC-Gal4-driven NF-YA RNAi cells compared with wild-type cells (Fig. 3G,H). Taken together, our results show that NF-Y is an essential regulator of mitochondrial biogenesis and lipid storage in ISCs and their progeny.

### NF-Y acts as a metabolic and proliferative brake through inhibiting mTORC1 signaling

**Fig. 4.**
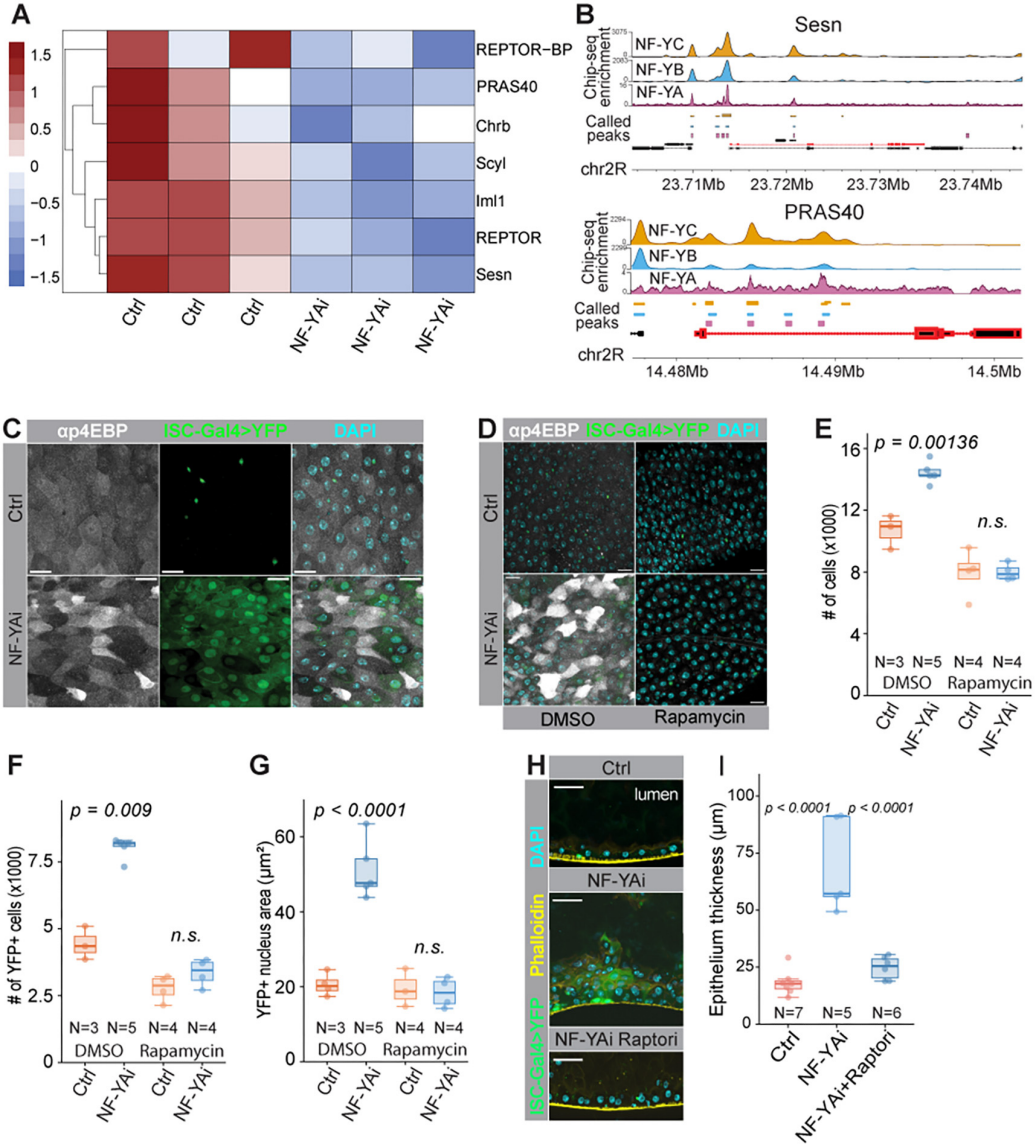
**NF-Y regulates proliferation and cell growth via mTORC1.** Heatmap of differentially expressed genes involved in the mTORC1 signaling pathway (GO database) from the mRNA sequencing experiment depicted in Fig. 3A. (B) Called peaks and track coverage of NF-YA, NF-YB and NF-YC ChIP-Seq on Sesn and PRAS40 loci (highlighted in red). The coverage track for the NF-YA subunit represents the fold change over control; coverage tracks for NF-YB and NF-YC represent control-normalized signals. (C) Representative images of esg-Gal4^ts^, Su(H)GBE-Gal80>NF-YA RNAi (KK) and control female midguts from the R4b region immunostained with α-p4EBP antibodies. Flies were kept at +29°C for 4 days. Scale bars: 20 μm. (D) Representative images of esg-Gal4^ts^, Su(H)GBE-Gal80>NF-YA RNAi (KK) and control female midguts from the R4b region kept on a holidic diet supplemented with either DMSO (control conditions) or 200 μM rapamycin. Scale bars: 20 μm. (E-G) Quantification of total cell numbers (E), YFP+ cell numbers (F) and YFP^+^ nuclear area (G) from the experiment depicted in D. (H) Representative cross-section images of esg-Gal4^ts^, Su(H)GBE-Gal80>NF-YA RNAi (KK), Raptor RNAi+NF-YA RNAi combination and control midgut from the R4b region; phalloidin stains F-actin. Scale bars: 20 μm. (I) Quantification of epithelium thickness from a cross-section from the experiment depicted in H. *P*-values for E and F were obtained by two-way ANOVA followed by Tukey's test. *P*-value in G was obtained by Welch analysis of variance (ANOVA) followed by the Games-Howell test. *P*-values in I were obtained by one-way ANOVA followed by Tukey's test. Box plots show median (line), interquartile range (box) and Tukey-style whiskers.

To understand how NF-Y regulates ISCs during nutrient adaptation, we identified NF-YA-regulated genes involved in nutrient sensing in our dataset. Indeed, we found several prominent negative regulators of the mTOR complex 1 (mTORC1) among the genes downregulated upon ISC-Gal4-driven NF-YA RNAi, including *PRAS40*, *Sestrin*, *Iml1* and *scylla*/*charybdis* (Fig. 4A). In addition, the expression of transcriptional effectors *REPTOR* and *REPTOR-BP* was also downregulated in these cells (Fig. 4A). Attenuation of Reptor is needed for the activation of the mTORC1-mediated transcriptional response (Tiebe et al., 2015). To determine whether these genes are directly regulated by the NF-Y transcription factor, we used the ModENCODE chromatin immunoprecipitation sequencing (ChIP-Seq) data (The modENCODE Consortium et al., 2010). Interestingly, the components of the NF-Y trimer showed identical binding to the gene regions of *Sestrin*, *PRAS40*, *scylla*/*charybdis* and *REPTOR* (Fig. 4B and Fig. S4A-C). These results suggest that NF-Y inhibits mTORC1 signaling in ISCs by increasing expression of genes involved in its negative-feedback regulation. To test this hypothesis, we stained midguts with antibodies against the phosphorylated form of the mTORC1 target initiation factor 4E-binding protein (p4EBP). Indeed, we observed very high p4EBP levels in the NF-YA LOF progenitors, indicating elevated mTORC1 activity relative to controls (Fig. 4C). To test whether the elevated mTORC1 activity is sufficient to drive the NF-YA loss of function-induced ISC proliferation and cell size increase, we inhibited mTORC1 through rapamycin feeding or genetically by knocking down the Regulatory-Associated protein of mTOR (Raptor) (Kim et al., 2002). Strikingly, we found that both rapamycin feeding and ISC-Gal4-driven knockdown of Raptor rescued the NF-YA loss of function-induced proliferation and cellular growth in ISCs (Fig. 4C-F and Fig. S4). In addition, knockdown of Raptor efficiently suppressed the epithelial thickening, increase in mitochondrial abundance, lipid droplet accumulation and epithelial disruption upon NF-YA loss of function (Fig. 4H,I and Fig. S5A-D). Taken together, our results show that NF-Y is an essential inhibitor of mTORC1 signaling in ISCs, thereby preventing overproliferation, progenitor growth and epithelial disruption during nutrient-adaptive midgut growth.

## DISCUSSION

Our study elucidates a previously unreported and unexpected role for NF-Y in *Drosophila* ISCs. NF-Y limits ISC proliferation and enterocyte size, reducing intestinal epithelial growth. Mechanistically, NF-Y regulates genes involved in the cell cycle, mitochondrial metabolism, lipid biosynthesis and mTORC1 signaling. Thus, NF-Y acts as a metabolic brake, protecting the intestinal epithelium from excessive growth during adaptation to increased nutrient intake.

NF-Y is a highly conserved trimeric eukaryotic transcription factor. It consists of the DNA-binding NF-YA and the regulatory subunits NF-YB and NF-YC. NF-Y plays key roles in many cell types and cellular processes. These range from early zygotic development to metabolism, the cell cycle and apoptosis (Li et al., 2018). Our results show that NF-Y has dual functions in ISCs. First, it restricts ISC proliferation by regulating the expression of key cell cycle regulators. Second, it reduces progenitor growth and the size of mature enterocytes. Interestingly, NF-YA knockdown in enteroblasts – the progenitors of enterocytes – did not increase cell growth in our conditions. This implies that the growth-restricting function of NF-Y in progenitors occurs already in ISCs. How does NF-Y mediate its function in ISCs to restrict progenitor growth and progeny size? A possible mechanism is through chromatin remodeling in ISCs. NF-Y interacts with co-repressors and co-activators in a cell- and context-specific manner, serving as a platform for chromatin remodeling proteins and integrating chromatin structure with transcriptional regulation (Oldfield et al., 2014). For example, NF-Y has previously been shown to regulate the chromatin landscape during early zygotic development. Depletion of maternally derived NF-YA in a mouse 2-cell embryo led to loss of open chromatin at promoter sites and developmental arrest (Lu et al., 2016). Future work is needed to resolve the exact role of NF-Y in chromatin remodeling in *Drosophila* ISCs. For example, identifying the binding partners of NF-Y in this setting could clarify how NF-Y regulates progenitor growth.

Beyond its role in early zygotic development, NF-Y has been implicated in regulating glucose and fatty acid metabolism in various cell types, including pancreatic β-cells, cardiomyocytes, hepatocytes and adipocytes (Cui et al., 2023; Liu et al., 2021; Lu et al., 2015; Zhang et al., 2018). In *Drosophila* ISCs, NF-Y represses mitochondrial metabolism and lipid accumulation by regulating gene expression involved in these processes. In addition, NF-Y restricts mTORC1 activity by regulating the expression of several pathway components. Regulation of mTORC1 signaling is a pivotal cellular mechanism for adjusting growth and anabolic metabolism to prevailing nutrient and energy availability (He et al., 2025). The finding that inhibiting mTORC1 activity in ISCs completely rescued the NF-Y loss-of-function phenotype suggests that the metabolic role of NF-Y is largely mediated by mTORC1. Thus, it would be interesting to know whether the same is true in other cellular contexts.

According to available mRNA sequencing data, NF-Y components are ubiquitously expressed across all midgut regions (Buchon et al., 2013). Yet our results show that NF-Y functions in the *Drosophila* midgut in a highly region-specific manner. NF-Y loss did not produce a noticeable phenotype in the R3 region, also known as the acidic copper cell region. R3 is populated by gastric stem cells (GSSCs), which are quiescent under homeostatic conditions but can be induced to proliferate by stress (Wang et al., 2014). However, little is known about how GSSCs sense and respond to nutritional inputs. For example, GSSCs exhibit lower mTORC1 activation in response to nutrients than ISCs in posterior regions, which could explain why these stem cells do not respond to NF-Y loss (Mattila et al., 2024). Future studies should address the region-specific role of NF-Y in regulating intestinal growth.

In ISCs, mTORC1 signaling primes the stem cells to differentiate toward the absorptive lineage, which, during differentiation, undergoes a massive increase in cellular size (Mattila et al., 2024; Quan et al., 2013). Constitutive activation of the mTORC1 pathway in ISCs, through loss of function of the tuberous sclerosis complex 1/2, leads to overgrowth, cell cycle arrest and a gradual loss of ISCs, possibly through delamination (Kapuria et al., 2012; Quan et al., 2013). Simultaneous activation of a pro-proliferative signal, such as a Notch loss-of-function condition, relieves the cell cycle arrest imposed by hyperactive mTORC1 in ISCs and leads to massive epithelial overgrowth (Kapuria et al., 2012; Quan et al., 2013). Thus, it is probable that NF-Y, in addition to regulating mTORC1 activity, also controls the activity of a pro-proliferative signal in ISCs. Future studies are needed to identify the full scope of NF-Y-regulated pathway activities in ISCs.

### Study limitations

Although our findings identify NF-Y as a key regulator of nutrient-adaptive epithelial growth, several questions remain unresolved. First, while our RNA-seq analysis together with published NF-Y ChIP-Seq datasets support a model in which NF-Y directly regulates multiple negative regulators of mTORC1 signaling, we have not experimentally validated NF-Y occupancy at these loci in ISCs. Therefore, we cannot exclude the possibility that some of the observed transcriptional changes are indirect consequences of NF-Y loss. Second, our data demonstrate that NF-YA protein levels are responsive to nutritional status, but the upstream mechanisms linking nutrient availability to NF-Y activity remain unknown. In particular, whether NF-Y is regulated by canonical nutrient-sensing pathways, such as insulin signaling, or by alternative metabolic inputs remains to be investigated. Finally, the growth-restrictive role of NF-Y described here was most prominent in specific regions of the adult midgut. The basis of this regional specificity remains unclear and may reflect local differences in nutrient sensing, stem cell behavior or metabolic state. Determining how NF-Y activity is integrated with region-specific signaling environments will be an important area for future study.

## MATERIALS AND METHODS

### *Drosophila* stocks and husbandry

Fly stocks used in the study: esg-Gal4, Tub-Gal80^ts^, Su(H)GBE-Gal80, 2xYFP (ISC-Gal4) (Zeng and Hou, 2015), NF-YA RNAi (VDRC 106132, Bloomington 25991), NF-YC RNAi (Bloomington 58234), Raptor-RNAi (Bloomington 31529), NF-YA-GFP (Bloomington 92638), Su(H)-Gal4, UAS-GFP; Tub-Gal80^ts^ (Zeng et al., 2010), MyO1A-Gal4, Tub-Gal80^ts^; UAS-GFP (Jiang et al., 2009) and Esg-ReDDM (Antonello et al., 2015). Fly stocks were maintained at +25°C, on nutritional medium containing agar 0.6% (w/v), malt 6.5% (w/v), semolina 3.2% (w/v), baker's yeast 1.8% (w/v), nipagin 2.4% and propionic acid 0.7%. In all experiments, flies were reared at +18°C and then transferred to +29°C to inhibit temperature-sensitive Gal80, thereby activating the UAS-GAL4 system in the midgut.

### Dietary treatments

Flies were kept on a chemically defined (holidic) diet (Piper et al., 2014), referred to as fed condition. For starvation, flies were kept on medium containing agar 0.6% (w/v), sucrose 2% (w/v), nipagin 2.4%, and propionic acid 0.7%. The pH of the starvation medium was adjusted to match the holidic diet (pH 6.8) using 5 M NaOH, thus excluding pH as a variable. For rapamycin feeding, the holidic diet was supplemented with 200 μM rapamycin (Thermo Scientific Chemicals, CAS #53123-88-9).

### Lifespan assay

Virgin ISC-Gal4>NF-YA RNAi and control female flies were collected and kept at +18°C for 3 days for midgut maturation. The flies were kept at a density of 10 animals per vial, containing holidic diet. The flies were then maintained at 29°C for the remainder of the experiment. The number of dead flies was scored, and the flies were transferred to fresh media every 3 days.

### Immunohistochemistry

The protocol for immunofluorescence staining was adapted from Micchelli (2014). Briefly, intestines were dissected in phosphate-buffered saline (PBS) and fixed in 8% PFA for 2 h. We used 8% PFA instead of the standard 4% to preserve the tissue during the long primary antibody incubation. The fixed tissue was then washed with 0.1% Triton X-100 in PBS and blocked with 1% BSA for 1 h. Subsequently, intestines were stained with primary antibodies at +4°C for 48 h. The primary antibodies used were anti-NF-YA (1:400) (a gift from Hideki Yoshida, Kyoto Institute of Technology, Japan), anti-β-galactosidase (1:400) (MP Biomedicals 0855976-CF), anti-Prospero (1:1000) (Developmental Studies Hybridoma Bank MR1A), anti-Dlg (1:50) (Developmental Studies Hybridoma Bank 4F3), anti-Armadillo (1:20) (Developmental Studies Hybridoma Bank N27A1), anti-p4EBP (1:400) (Cell Signaling 2855), anti-Horseradish Peroxidase HRP (1:200) (Jackson ImmunoResearch Laboratories 2338967), anti-TOM20 1:400 (BD Biosciences 612278), anti-Innexin 2 (1:1000) (a gift from Alexander Borst, Max Planck Institute, Martinsried, Germany) (Ammer et al., 2022), anti-Pdm1 (1:25) (Developmental Studies Hybridoma Bank Nub 2D4) and Phalloidin (1:300) (Invitrogen A12380). Samples were mounted in Vectashield with DAPI (Vector Laboratories).

### EdU feeding and Click-it-assay

EdU (Sigma-Aldrich 95740-26-4) was added to holidic food at a concentration of 0.2 mg/ml. Flies were fed the medium containing EdU for 16 h, then dissected in PBS and fixed for 30 min in 8% PFA. After fixation, samples were washed with 0.1% Triton X-100 in PBS and incubated for 20 min in the click assay cocktail (Baseclick, ClickTech EdU Proliferation Kit for High-throughput Screening). The cocktail solution was removed, and samples were washed in PBS containing 0.1% Triton X-100 for 1 h. Subsequently, samples were mounted in Vectashield with DAPI (Vector Laboratories).

### Lipidtox staining

Midguts were dissected in Shields and Sang M3 medium (Sigma-Aldrich S8398) and fixed in 8% PFA for 30 min. The midguts were washed in PBS and stained with HCS LipidTOX Deep Red Neutral Lipid Stain 1:400 (Invitrogen H34477) in PBS for 30 min. The midguts were washed in PBS and mounted in Vectashield with DAPI (Vector Laboratories).

### Measurement of epithelial thickness

To quantify the multilayering phenotype, cross-sections of the R4b midgut region were imaged with the Aurox Clarity spinning-disk confocal microscope. Epithelial thickness was measured in ImageJ by manually drawing a perpendicular line, stretching from the inner boundary of the visceral muscle layer to the apical brush border of ECs. Line placement was based on the F-actin signal (phalloidin), which labels both the visceral muscle and the apical brush border of cells, with DAPI and ISC-Gal4-driven GFP (in NF-YA RNAi case) used as additional landmarks to distinguish the basal and apical epithelial boundaries. The length of each line was recorded as epithelial thickness (μm). The epithelium thickness per midgut corresponds to the average of nearly 15 measurements.

### Microscopy and image processing

Fixed and immunostained midguts were mounted between a microscope slide and a coverslip using 0.12 μm spacers. The samples were imaged using the Aurox Clarity spinning-disk confocal or the Leica SP8 confocal microscope. Images were further processed in ImageJ and segmented using Stardist as previously described (Viitanen et al., 2021).

### Transmission electron microscopy

Midguts were dissected in PBS and fixed in 1% glutaraldehyde, 2% PFA, 2 mM CaCl2 and 100 mM cacodylate buffer (pH 7.5) for 1 h. After dehydration, the samples were embedded in Epon resin. Samples were imaged on a Jeol JEM-1400 transmission electron microscope.

### FACS and RNA sequencing

The protocols for FACS and RNA sequencing were adapted from Dutta et al. (2013). Three independent samples from the control and NF-YA RNAi lines, driven by the ISC-Gal4 driver, were processed in parallel. Female flies were kept on holidic diet for 3 days at +29°C, and then the intestines were dissected and processed into cell lysates. After dissociation, cells were filtered through a 40 μm filter and FACS-isolated based on YFP signal. RNA was extracted using the ArcturusTM PicoPureTM RNA isolation Kit (Thermo Fisher Scientific 3032689). RNA concentration and quality were assessed using Agilent Bioanalyzer, followed by sequencing on the AVITI High Output (two-end reads, length 75 bp) platform. Raw RNA-seq data were processed using nf-core/rnaseq pipeline (v 3.18.0) (Ewels et al., 2020). Reads were trimmed using Trim Galore (v 0.6.10) and subsequently aligned with STAR (v 2.7.11b) to the *D. melanogaster* reference genome (FlyBase r6.62). Salmon (v 1.10.3) was used to obtain transcript-level counts. All processing steps were orchestrated with Nextflow (v 24.10.3) and executed within Singularity containers (v 3.18.0). Heatmaps were generated with tidyheatmaps (v 0.2.1) (https://jbengler.github.io/tidyheatmaps/authors.html#citation). The raw and processed sequencing data have been deposited in GEO under accession number GSE317617. To identify significantly enriched pathways, gene set enrichment analysis (GSEA) included KEGG, WikiPathways, Reactome and Gene Ontology (GO) databases, and was executed using the clusterProfiler package (version 4.12.6) (Wu et al., 2021) in R/Bioconductor. Pathways with an adjusted *P*<0.05 with Benjamini–Hochberg correction were considered significantly enriched.

### ChIP-Seq

Browser Extensible Data (BED) files and control-normalized bigWig files of NF-YA (GSE257055), NF-YB (GSE256859), and NF-YC (GSE256684) were obtained and paired with respective input controls (ENCSR177ZME, ENCSR640GRU and ENCSR877IXV) from the ENCODE portal (https://www.encodeproject.org/) (ENCODE Project Consortium, 2012). Peaks and coverage tracks were visualized with karyoploteR (v1.30.0) (Gel and Serra, 2017). Coverage tracks for NF-YB and NF-YC represent control-normalized signal, and the NF-YA coverage track represents the fold change over control. All signal tracks were generated by the ENCODE uniform processing pipeline. Called peaks correspond to optimal IDR-thresholded peak calls from the ENCODE ChIP-Seq processing pipeline, representing genomic regions where ChIP signal significantly exceeds matched input background across replicates.

### Statistical analysis

Statistical analysis was performed using R/Bioconductor and GraphPad Prism. Homogeneity of variances was assessed using Levene's test, and normality was evaluated using the Shapiro–Wilk test. For comparisons between two groups, either an unpaired Student's *t*-test or the Wilcoxon rank-sum test was used, depending on variance homogeneity and data normality. For multiple group comparisons, one-way ANOVA with Tukey's post hoc test was used. For multiple comparisons with two variables (diet), data were analyzed using either two-way ANOVA followed by Tukey's post-hoc test or Welch's ANOVA followed by the Games–Howell post-hoc test. Survival curves were analyzed using the log-rank (Mantel–Cox) test. The exact tests, sample sizes, and P-values for each experiment are provided in the figures and their figure legends.

## Supplementary Material



10.1242/develop.205643_sup1 Supplementary information

Table S1. List of differentially expressed genes in NF-YA-deficient ISCs.
