# Supplementary material for: Nuclear Factor Y controls nutrient-adaptive epithelial growth by regulating mTOR in the Drosophila midgut
Source: Development. 2026 Jul 13;153(13):dev205643. doi: 10.1242/dev.205643 (PMC13405225; doi:10.1242/dev.205643)
Supplement: Supplementary information [file develop-153-205643-s1.pdf]

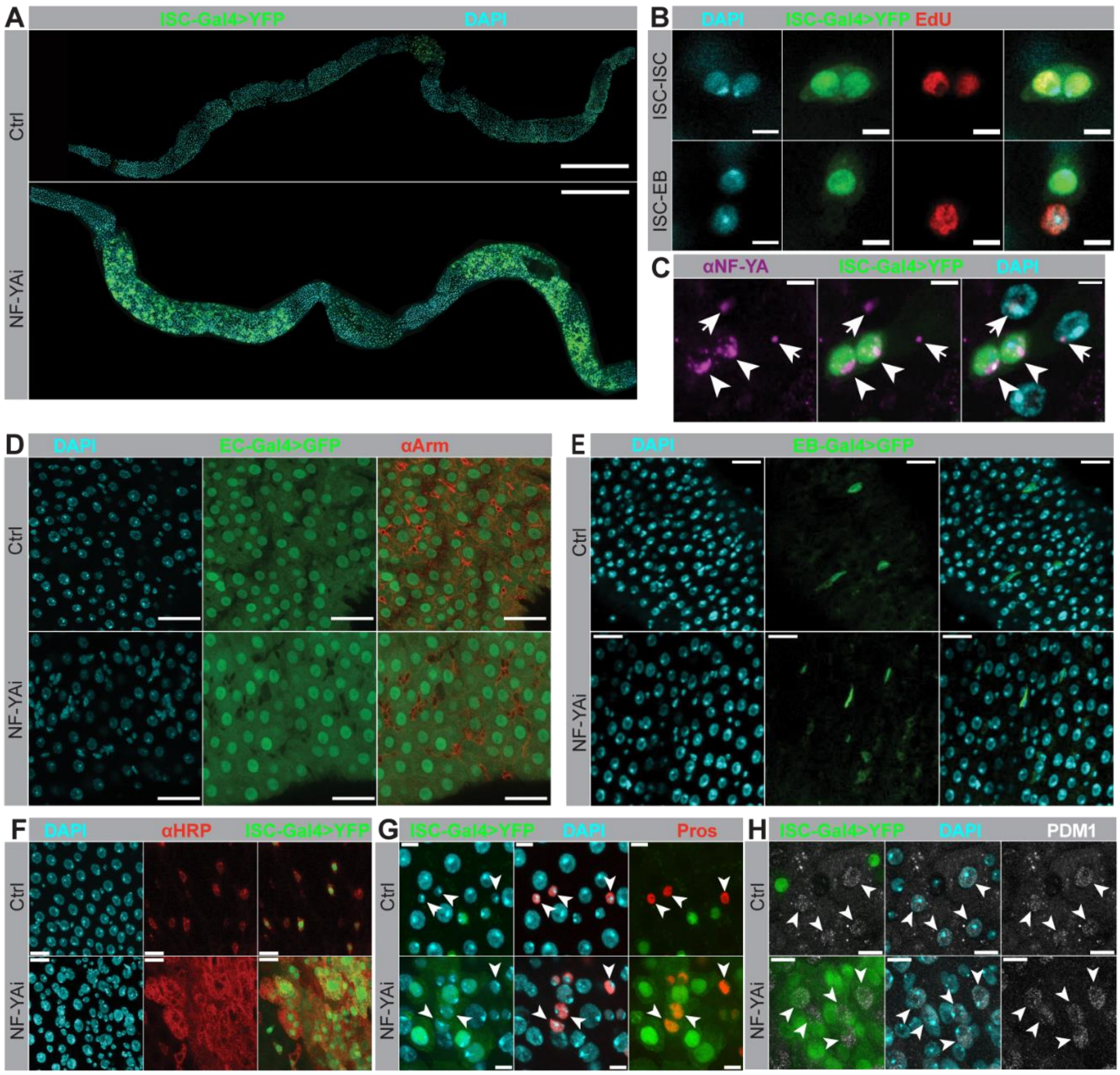

Fig. S1. Related to the main Figure 1.

(A) Representative images of esg-Gal4^{ts}, Su(H)GBE-Gal80 > NF-YA RNAi (Trip), and control female midguts. Scale bars: 500 µm. (B) Representative images of esg-Gal4^{ts}, Su(H)GBE-Gal80 > YFP ISC-ISC and ISC-EB cell pairs showing EdU incorporated chromatin. Scale bars: 5 µm. (C) Representative images of esg-Gal4^{ts}, Su(H)GBE-Gal80 midguts immunostained with α-NF-YA antibodies. Arrowheads point to YFP+ ISCs, and arrows point to ECs. Scale bars: 15 µm. (D) Representative images of Myo1A-Gal4^{ts} > NF-YA RNAi (KK) and control midguts from the R4b region immunostained with α-Armadillo antibodies. Scale bars: 30 µm. (E) Representative images of Su(H)-Gal4^{ts} > NF-YA RNAi (KK) and control midguts from the R4b region. Scale bars: 20 µm. (F) Representative images of esg-Gal4^{ts}, Su(H)GBE-Gal80 > NF-YA RNAi (KK), and control female midguts from the R4b region immunostained with α-HRP antibodies. Scale bars: 20 µm. (G) Representative images of esg-Gal4^{ts}, Su(H)GBE-Gal80 > NF-YA RNAi (KK), and control female midguts from the R4b region immunostained with α-Prospero antibodies. Arrowheads point to EEs. Scale bars: 15 µm. (H) Representative images of esg-Gal4^{ts}, Su(H)GBE-Gal80 > NF-YA RNAi (KK), and control female midguts from the R4b region immunostained with α-Pdm1 antibodies. Arrowheads point to ECs. Scale bars: 15 µm.

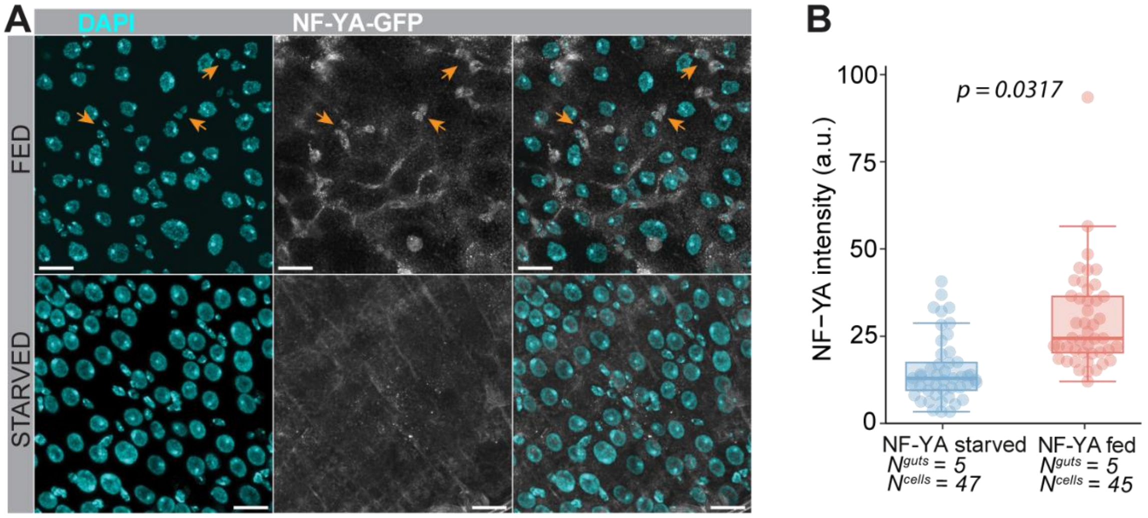

Fig. S2. Related to the main Figure 2.

(A) Representative images of NF-YA-GFP localization in fed and starved conditions. Arrows point to small nuclear cells. Scale bars: 15 µm. (B) Quantification of the NF-YA-GFP intensity in the nucleus of small nuclear cells from the experiment depicted in A. P-value in B was obtained by the Wilcoxon rank-sum test. Box plots show median, interquartile range, and Tukey-style whiskers.

Development • Supplementary information

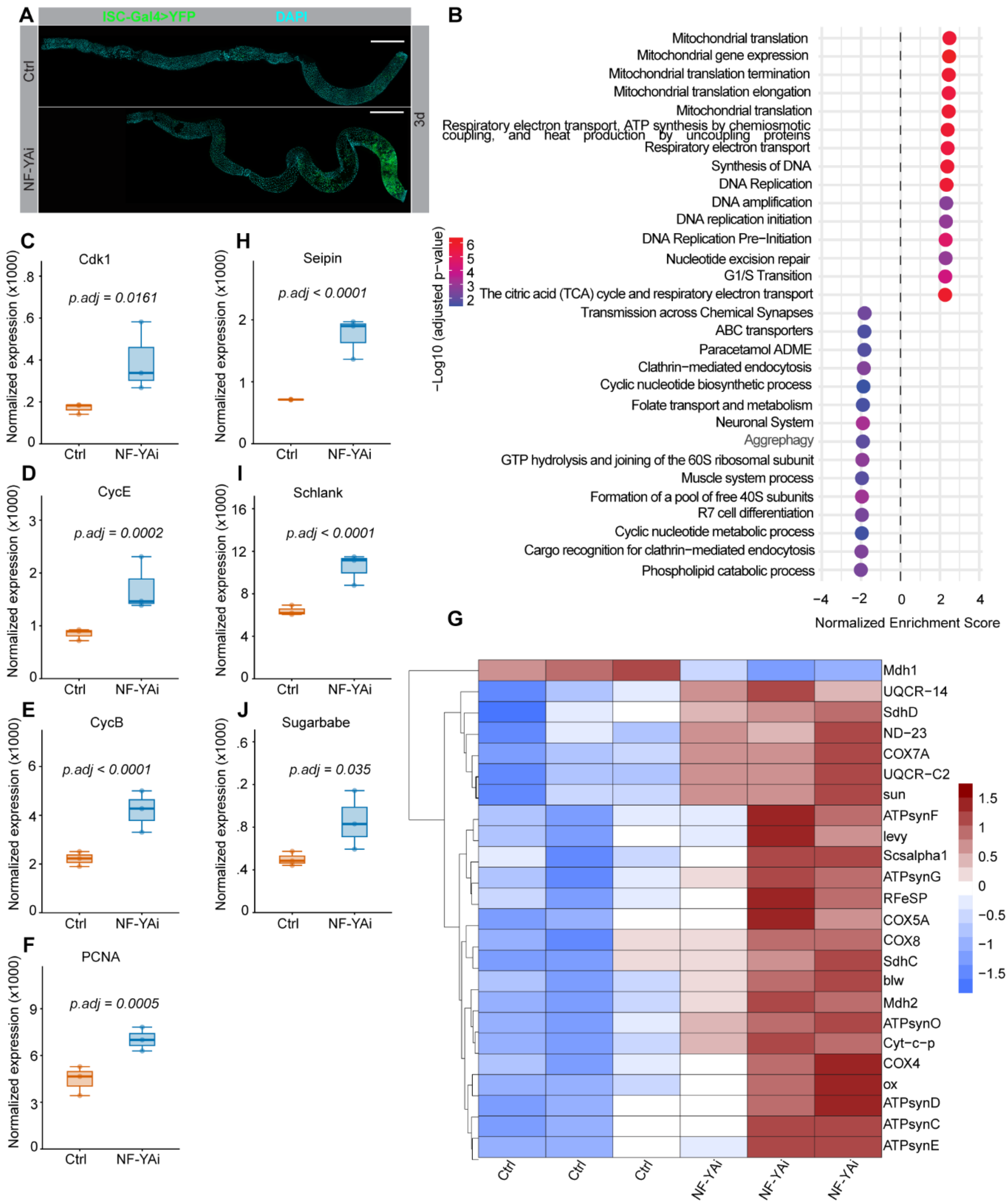

Fig. S3. Related to the main Figure 3.

(A) Representative images of esg-Gal4^{ts}, Su(H)GBE-Gal80 > NF-YA RNAi (KK), and control female midguts after 3 days at +29°C. Scale bars: 500 μm. (B) Gene-set enrichment analysis (GSEA) from the mRNA sequencing experiment depicted in Figure 3A. The analysis includes gene set annotations from the Gene Ontology (GO), Kyoto Encyclopedia of Genes and Genomes (KEGG), WikiPathways (WP) and Reactome databases. (C-F) Normalized mRNA expressions of Cdk1 (C), CycE (D), CycB (E), and PCNA (F). (G) Heatmap representing differentially expressed genes of the tricarboxylic acid cycle and the electron transport chain (GO database) from the RNA sequencing experiment depicted in Figure 3A. (H-J) Normalized mRNA expressions of Seipin (H), Schlank (I), and Sugarbabe (J). Box plots show median, interquartile range and Tukey-style whiskers.

Development • Supplementary information

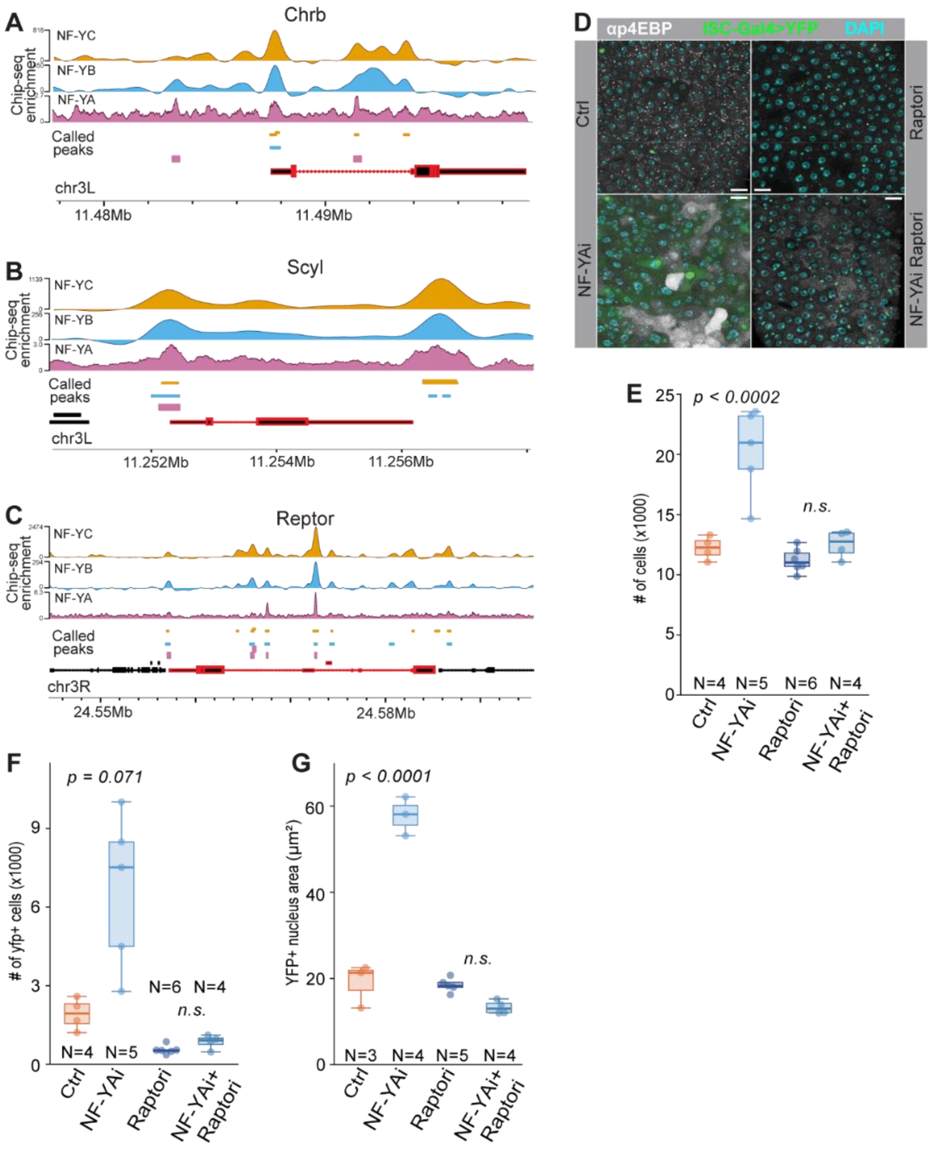

Fig. S4. Related to the main Figure 4.

(A-C) Called peaks and track coverage of NF-YC, NF-YB, and NF-YA ChIP-Seq on Chrb (A), Scyl (B), and Reptor (C). The coverage track for the NF-YA subunit represents the fold change over control; coverage tracks for NF-YB and NF-YC represent control-normalized signals. (D) Representative images of esg-Gal4^{ts}, Su(H)GBE-Gal80 > NF-YA RNAi (KK), > Raptor RNAi, > Raptor RNAi + NF-YA RNAi combination, and control female midguts from the R4b region immunostained with α-p4EBP antibodies. Scale bars: 15 µm. (E-G) Quantification of total cell numbers (E), YFP+ cell numbers (F), and YFP+ nuclear area (G) from the experiment depicted in (D). P-value in (F) was obtained by Welch analysis of variance (ANOVA) followed by the Games-Howell test. P-values in (E) and (G) were obtained by two-way ANOVA followed by Tukey's test. Box plots show median, interquartile range, and Tukey-style whiskers.

Development • Supplementary information

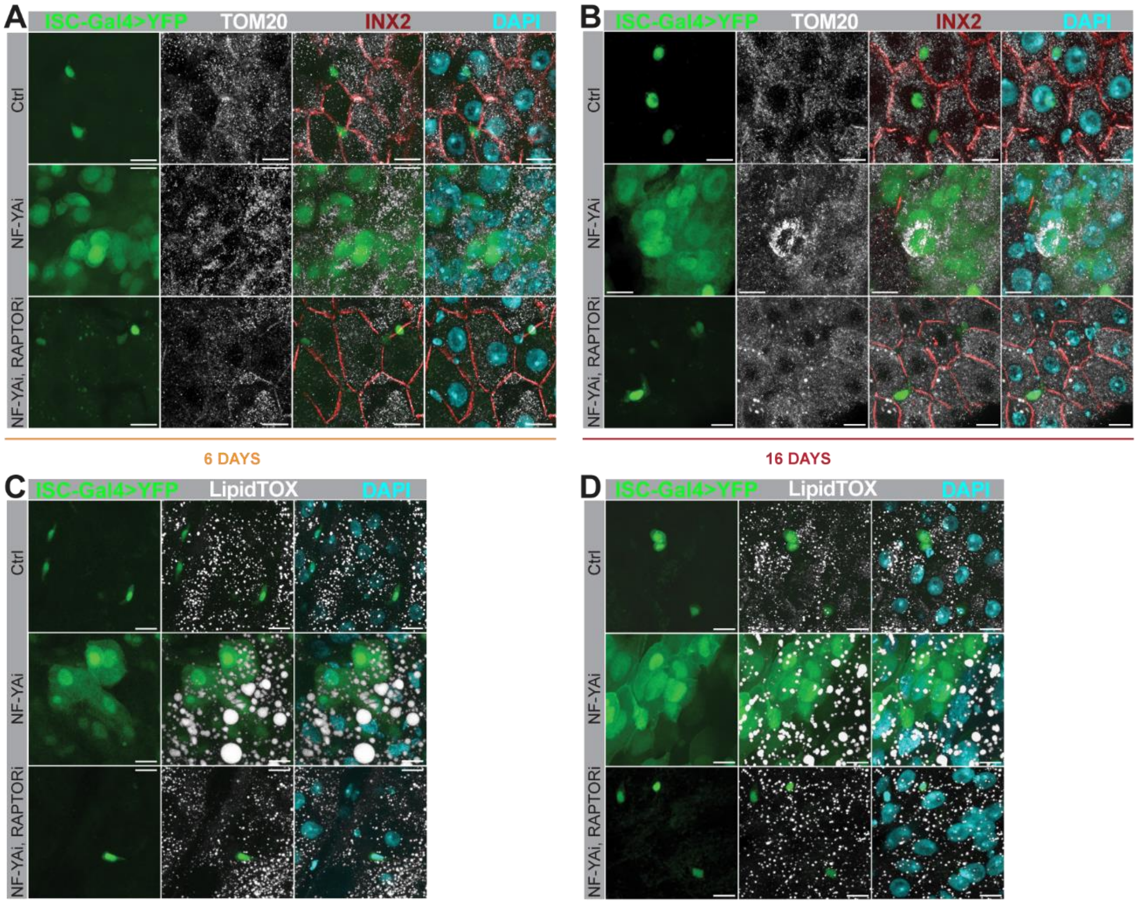

Fig. S5. Related to the main Figure 4.

(A, B) Representative images of 6d-old (A) and 16d-old (B) esg-Gal4^{ts}, Su(H)GBE-Gal80 > NF-YA RNAi (KK), > Raptor RNAi + NF-YA RNAi combination, and control female midguts from the R4b region immunostained with α-Tom20 and α-Innexin2 antibodies. Innexin2 is absent in the NF-YA knockdown condition, indicating loss of gap junctions and epithelial integrity. Scale bars: 10 μm. (C, D) Representative images of 6d-old (C) and 16d-old esg-Gal4^{ts}, Su(H)GBE-Gal80 > NF-YA RNAi (KK), > Raptor RNAi + NF-YA RNAi combination, and control female midguts from the R2b stained with LipidTox. Scale bars: 10 μm.

Table S1. List of differentially expressed genes in NF-YA-deficient ISCs.

Available for download at
https://journals.biologists.com/dev/article-lookup/doi/10.1242/dev.205643#supplementary-data

Development • Supplementary information
