# Supplementary material for: Nuclear Factor Y controls nutrient-adaptive epithelial growth by regulating mTOR in the Drosophila midgut
Source: Development. 2026 Jul 13;153(13):dev205643. doi: 10.1242/dev.205643 (PMC13405225; doi:10.1242/dev.205643)
Supplement: Supplementary file 1 [file develop-153-205643_review_history.pdf]

Nuclear Factor Y controls nutrient-adaptive epithelial growth by regulating mTOR in the *Drosophila* midgut

Tetiana Strutynska, Onur Deniz and Jaakko Mattila
DOI: 10.1242/dev.205643

Editor: Irene Miguel-Aliaga

Review timeline
Original submission: 6 March 2026
Editorial decision: 22 March 2026
First revision received: 12 June 2026
Accepted: 29 June 2026

Original submission

First decision letter

MS ID#: dev.205643

MS TITLE: Nuclear Factor Y controls nutrient-adaptive epithelial growth by regulating mTOR in the Drosophila midgut

AUTHORS: Tetiana Strutynska, Onur Deniz and Jaakko Mattila

Dear Dr Mattila,

I have now received all the referees' reports on the above manuscript, and have reached a decision. The referees' comments are appended below.

As you will see, the referees express considerable interest in your work, but have some significant criticisms and recommend a substantial revision of your manuscript before we can consider publication. If you are able to revise the manuscript along the lines suggested, which may involve further experiments, I will be happy receive a revised version of the manuscript. Your revised paper will be re-reviewed by one or more of the original referees, and acceptance of your manuscript will depend on your addressing satisfactorily the reviewers' major concerns. Please also note that Development will normally permit only one round of major revision. If it would be helpful, you are welcome to contact us to discuss your revision in greater detail. Please send us a point-by-point response indicating your plans for addressing the referees' comments, and we will look over this and provide further guidance.

Please attend to all of the reviewers' comments and ensure that you upload both a 'clean' version of your Word file, along with a highlighted version clearly showing where you have made changes in the revised manuscript. Please avoid using 'Tracked changes' in Word files as these are lost in PDF conversion. I should be grateful if you would also provide a point-by-point response detailing how you have dealt with the points raised by the reviewers in the 'Response to Reviewers' box. If you do not agree with any of their criticisms or suggestions please explain clearly why this is so.

Reviewer 1

The study "Nuclear Factor Y controls epithelial growth by regulating mTOR in the Drosophila midgut" by Tetiana Strutynska; Onur Deniz; Jaakko Mattila identifies the Drosophila NF-Y ortholog, as an regulator of intestinal stem cells (ISC) proliferation and differenciation. The authors suggest

© 2026. Published by The Company of Biologists under the terms of the Creative Commons Attribution License (https://creativecommons.org/licenses/by/4.0/).

that NF-Y is an essential inhibitor of mTORC1 signaling in ISCs, thereby preventing overproliferation, progenitor growth, and epithelial disruption in nutrient-rich conditions.

The experiments are well designed. The general characterization of NF-Y lof in ISC, the transcriptional approach validated in vivo by the described effects on mitochondrial biogenesis and lipid storage and the genetic epistasis with TORC1 using both RNAi or rapamycin treatments are convincing. Overall, the paper presents interesting lines of evidence that support the proposed model and is relevant for a broad audience.

Minor points:

- The rescue of NF-Y RNAi effects by starvation is quite impressive. Direct inhibition of TORC1 in this context is probably the main driver of the phenotype but is there any evidence of a direct regulation of NF-Y levels by starvation? Is NP-Y accumulating in starvation conditions (qPCR or immuno-staining)? Is NP-Y a target of the Insulin signalling/ FOXO pathway? What could be regulating NF-Y transcriptional activity?

- Line 125: Figure 1K-N should be Figure 2D-G

Reviewer 2

SUMMARY OF THE ADVANCE MADE IN THIS PAPER AND ITS POTENTIAL SIGNIFICANCE TO THE FIELD

In this manuscript, Strutynska et al. investigate the role of the transcription factor NF-Y in regulating intestinal epithelial growth in the Drosophila midgut. The authors present evidence that NF-Y restricts intestinal stem cell (ISC) proliferation, progenitor growth, and epithelial expansion, and propose that this occurs through modulation of mTORC1 signaling. The study integrates genetic perturbations, lineage tracing, transcriptomic profiling, ultrastructural analysis, and pharmacological approaches to support a model in which NF-Y functions as a metabolic and proliferative brake.
Overall, this is a well-executed and clearly written study addressing an important question in tissue homeostasis. The integration of stem cell biology with metabolic regulation is a particular strength, and many of the experimental observations are convincing.

SUGGESTIONS TO AUTHORS
[Major concerns]
(1) A conceptual concern relates to the use and interpretation of the term "nutrient-rich conditions" throughout the manuscript. The central claim is that NF-Y limits excessive epithelial growth specifically under "nutrient-rich conditions." However, the experimental framework supporting this conclusion relies primarily on a comparison between a chemically defined holidic diet and a 2% sucrose diet. The latter lacks amino acids and other essential nutrients and is therefore more appropriately interpreted as a starvation or near-starvation condition, rather than a physiologically relevant "nutrient-poor" state. As a consequence, it remains unclear whether NF-Y specifically buffers excessive growth under nutrient-rich conditions, or whether the apparent suppression of the NF-Y loss-of-function phenotypes under the 2% sucrose condition may simply reflect the well-established reduction of mTORC1 activity during starvation. This distinction is important because it directly affects the conceptual advance of the study. To address this issue, the authors should either provide additional experimental evidence using graded dietary conditions, or substantially revise the wording throughout the manuscript to avoid overinterpretation. In particular, the use of terms such as "nutrient-rich conditions" should be reconsidered and replaced with more neutral descriptions (e.g., "fed conditions"), and the conclusions should be reframed accordingly in the Abstract, Results, and Discussion.

(2) The mechanistic link between NF-Y and mTORC1 signaling is not fully established. The authors propose that NF-Y restrains mTORC1 activity by regulating transcription of prominent negative regulators of mTORC1, such as Iml1, Sestrin, and Pras40. While the RNA-seq data and p4EBP staining are consistent with this model, the evidence remains largely correlative. The manuscript does not establish whether these genes are direct transcriptional targets of NF-Y, nor does it examine enrichment of NF-Y binding motifs or provide evidence of NF-Y occupancy at relevant loci. Additional analyses, such as promoter motif enrichment or targeted binding assays, would strengthen this aspect of the study. Alternatively, the authors should moderate the mechanistic

© 2026. Published by The Company of Biologists under the terms of the Creative Commons Attribution License (https://creativecommons.org/licenses/by/4.0/).

claims and more clearly distinguish between direct and indirect regulatory effects, as the current wording risks overstating the level of mechanistic resolution.

(3) The experiments in which mTORC1 activity is reduced, either pharmacologically by rapamycin or genetically via Raptor knockdown, are a strong aspect of the study and provide compelling evidence that elevated mTORC1 activity contributes to the NF-Y loss-of-function phenotype. However, the extent to which mTORC1 accounts for the full spectrum of observed phenotypes is not entirely clear. It would be helpful to clarify whether all aspects of the phenotype, including mitochondrial expansion, lipid accumulation, and epithelial multilayering, are equally sensitive to mTORC1 inhibition, or whether some features persist. Providing additional quantification or refining the interpretation would strengthen the conclusions and avoid overgeneralization.

(4) The region-specific nature of the phenotype is intriguing but remains largely unexplored. The restriction of NF-Y loss-of-function effects to specific midgut regions suggests underlying spatial heterogeneity in signaling or metabolic state. A more detailed discussion of potential mechanisms, even if speculative, would enhance the depth of the manuscript.

(5) The identification of enlarged YFP-positive cells as undifferentiated progenitors is based primarily on HRP staining. Given the substantial changes in cell size and epithelial organization, it would be important to further validate cell identity using additional lineage or differentiation markers, such as Prospero and Pdm1.

[Minor points]
(i) The authors may also consider reflecting the nutrient-dependent aspect of their findings more explicitly in the title, as this represents a central component of the study.

(ii) L125-126: The description of NF-Y as an "obligate inhibitor" appears too strong and should be softened.

(iii) L262: The fixation conditions used for immunostaining (8% PFA) are relatively strong and would benefit from a brief justification.

First revision

<u>Author response to reviewers' comments</u>

Reviewer 1: The study "Nuclear Factor Y controls epithelial growth by regulating mTOR in the Drosophila midgut" by Tetiana Strutynska; Onur Deniz; Jaakko Mattila identifies the Drosophila NF-Y ortholog, as an regulator of intestinal stem cells (ISC) proliferation and differenciation. The authors suggest that NF-Y is an essential inhibitor of mTORC1 signaling in ISCs, thereby preventing overproliferation, progenitor growth, and epithelial disruption in nutrient-rich conditions.
The experiments are well designed. The general characterization of NF-Y lof in ISC, the transcriptional approach validated in vivo by the described effects on mitochondrial biogenesis and lipid storage and the genetic epistasis with TORC1 using both RNAi or rapamycin treatments are convincing. Overall, the paper presents interesting lines of evidence that support the proposed model and is relevant for a broad audience.

We thank the reviewer for the positive overall evaluation of our study.

Minor points:
1. -The rescue of NF-Y RNAi effects by starvation is quite impressive. Direct inhibition of TORC1 in this context is probably the main driver of the phenotype but is there any evidence of a direct regulation of NF-Y levels by starvation? Is NP-Y accumulating in starvation conditions (qPCR or immuno-staining)?

© 2026. Published by The Company of Biologists under the terms of the Creative Commons Attribution License (https://creativecommons.org/licenses/by/4.0/).

This is a very interesting question, and addressing it experimentally would significantly strengthen our manuscript. Thus, we have explored the regulation of NF-YA by using an available fly line harboring C-terminally GFP-tagged NF-YA, whose expression is regulated by endogenous promoter. Indeed, we observed increased levels of NF-YA protein under fed compared with starved conditions. This is in line with our model, which posits that NF-Y activity is necessary under nutrient-rich conditions to restrict mTORC1 activity.

2. Is NP-Y a target of the Insulin signalling/ FOXO pathway?

Gene regulation by dFoxo has been studied extensively. For example, (Alic et al., 2011) *identified over 700 direct transcriptional targets of the dFoxo transcription factor from adult flies. None of the NF-Y components were among the list of genes regulated by dFoxo. However, NF-YA was shown to be expressed in the larval fat body, where it regulates lipid biosynthesis by repressing Lip-4 gene expression, strongly suggesting a nutrient-responsive function* (Yoshioka et al., 2022). *Interestingly, Lip-4 gene expression is activated by dFoxo in the larval fat body* (Vihervaara and Puig, 2008). *Together, these data suggest that NF-Y and dFoxo regulate the same nutrient-responsive genes but in opposing directions in the fat body. How the interplay between dFoxo and NF-Y is regulated, in general and specifically in intestinal stem cells, is an interesting question but is beyond the scope of the current study.*

3. What could be regulating NF-Y transcriptional activity?

Our findings that NF-YA protein levels are responsive to nutrients (see above point #1) provide strong evidence that NF-YA transcription is regulated by nutrients. The exact mechanism underlying this regulation is an intriguing question for future studies. See also our 'Study limitations' section.

4. -Line 125: Figure 1K-N should be Figure 2D-G

We have corrected this mistake.

Reviewer 2: SUMMARY OF THE ADVANCE MADE IN THIS PAPER AND ITS POTENTIAL SIGNIFICANCE TO THE FIELD

In this manuscript, Strutynska et al. investigate the role of the transcription factor NF-Y in regulating intestinal epithelial growth in the Drosophila midgut. The authors present evidence that NF-Y restricts intestinal stem cell (ISC) proliferation, progenitor growth, and epithelial expansion, and propose that this occurs through modulation of mTORC1 signaling. The study integrates genetic perturbations, lineage tracing, transcriptomic profiling, ultrastructural analysis, and pharmacological approaches to support a model in which NF-Y functions as a metabolic and proliferative brake.

Overall, this is a well-executed and clearly written study addressing an important question in tissue homeostasis. The integration of stem cell biology with metabolic regulation is a particular strength, and many of the experimental observations are convincing.

We appreciate the overall positive feedback on our study.

SUGGESTIONS TO AUTHORS

[Major concerns]

(1) A conceptual concern relates to the use and interpretation of the term "nutrient-rich conditions" throughout the manuscript. The central claim is that NF-Y limits excessive epithelial growth specifically under "nutrient-rich conditions." However, the experimental framework supporting this conclusion relies primarily on a comparison between a chemically defined holidic diet and a 2% sucrose diet. The latter lacks amino acids and other essential nutrients and is therefore more appropriately interpreted as a starvation or near-starvation condition, rather than a physiologically relevant "nutrient-poor" state. As a consequence, it remains unclear whether NF-Y specifically buffers excessive growth under nutrient-rich conditions, or whether the apparent suppression of the NF-Y loss-of-function phenotypes under the 2% sucrose condition may simply reflect the well-established reduction of mTORC1 activity during starvation. This distinction is important because it directly affects the conceptual advance of the study. To address this issue, the authors should either provide additional experimental evidence using graded dietary conditions, or substantially revise the wording throughout the manuscript to avoid overinterpretation. In particular, the use of

© 2026. Published by The Company of Biologists under the terms of the Creative Commons Attribution License (https://creativecommons.org/licenses/by/4.0/).

terms such as "nutrient-rich conditions" should be reconsidered and replaced with more neutral descriptions (e.g., "fed conditions"), and the conclusions should be reframed accordingly in the Abstract, Results, and Discussion.

The reviewer is concerned about the validity of the interpretation that NF-Y functions to restrict excessive tissue growth under 'nutrient-rich' conditions. We agree that the term 'nutrient-rich' is unambiguous, and we have reframed our conclusions with more appropriate terms such as 'nutrient-adaptive'.

In addition, we have now conducted an additional experiment in which, as suggested by the reviewer, we used graded dietary conditions (100%- 75%- 50%- 25%- 10% holidic diet) and monitored intestinal epithelial thickening in ISC-Gal4 > NF-YA RNAi animals. Indeed, the phenotype becomes less severe with diluted diets and is completely diminished at the 10% holidic diet. These new data strengthen our conclusion that NF-Y acts as a growth-restricting factor under nutrient-adaptive growth conditions. These data are now included in Figure 2D.

Finally, we noticed an unintentional mislabeling of Figure 2D-G, where we had actually used a 10% holidic diet dilution instead of the 2% sucrose diet (starvation, as it was mislabeled in the original manuscript). Thus, the phenotype was originally compared between a 100% holidic diet (nutrient-rich) and a 10% holidic diet (nutrient-poor). We have corrected this mistake.

(2) The mechanistic link between NF-Y and mTORC1 signaling is not fully established. The authors propose that NF-Y restrains mTORC1 activity by regulating transcription of prominent negative regulators of mTORC1, such as Iml1, Sestrin, and Pras40. While the RNA-seq data and p4EBP staining are consistent with this model, the evidence remains largely correlative. The manuscript does not establish whether these genes are direct transcriptional targets of NF-Y, nor does it examine enrichment of NF-Y binding motifs or provide evidence of NF-Y occupancy at relevant loci. Additional analyses, such as promoter motif enrichment or targeted binding assays, would strengthen this aspect of the study. Alternatively, the authors should moderate the mechanistic claims and more clearly distinguish between direct and indirect regulatory effects, as the current wording risks overstating the level of mechanistic resolution.

The reviewer asks for evidence that NF-Y directly regulates mTORC1 signaling by binding to the promoters of prominent mTORC1 regulators. We have explored this question by using ChIP-seq data from the modENCODE project. Indeed, we noticed that the components of the NF-Y trimer all share significantly enriched binding profiles within the gene regions of several mTORC1 negative regulators, such as Sestrin, Pras40, Scy and Chrb. Together with our RNA-seq data, these findings strongly suggest a direct regulatory effect of NF-Y on these genes. We have now included this new data in Figure 4B and Figure S4A-C. See also 'Study limitations' section.

(3) The experiments in which mTORC1 activity is reduced, either pharmacologically by rapamycin or genetically via Raptor knockdown, are a strong aspect of the study and provide compelling evidence that elevated mTORC1 activity contributes to the NF-Y loss-of-function phenotype. However, the extent to which mTORC1 accounts for the full spectrum of observed phenotypes is not entirely clear. It would be helpful to clarify whether all aspects of the phenotype, including mitochondrial expansion, lipid accumulation, and epithelial multilayering, are equally sensitive to mTORC1 inhibition, or whether some features persist. Providing additional quantification or refining the interpretation would strengthen the conclusions and avoid overgeneralization.

We have now performed additional experiments to clarify which part of the NF-Y LOF phenotype is sensitive to mTORC1 inhibition. We have used a double knockdown of NF-YA and Raptor and monitored the phenotype over time. We have used antibodies against TOM-20 and LipidTox to visualize mitochondria and lipid droplets, respectively, under NF-YA/Raptor double-knockdown conditions. To visualize epithelial integrity, we have measured epithelial thickness and used antibodies against Innexin alpha. Our results show that the accumulation of mitochondria and lipid droplets, as well as the epithelial disruption upon NF-YA loss, is completely rescued by Raptor RNAi. These data are in line with the conclusion that NF-Y's metabolic role is largely mediated by mTORC1. The new data is included in Figure 4H,I and S5.

© 2026. Published by The Company of Biologists under the terms of the Creative Commons Attribution License (https://creativecommons.org/licenses/by/4.0/).

(4) The region-specific nature of the phenotype is intriguing but remains largely unexplored. The restriction of NF-Y loss-of-function effects to specific midgut regions suggests underlying spatial heterogeneity in signaling or metabolic state. A more detailed discussion of potential mechanisms, even if speculative, would enhance the depth of the manuscript.

We have included a paragraph in the discussion about the region-specific nature of NF-Y in regulating midgut growth (Lines 257-266). See also 'Study limitations' section.

(5) The identification of enlarged YFP-positive cells as undifferentiated progenitors is based primarily on HRP staining. Given the substantial changes in cell size and epithelial organization, it would be important to further validate cell identity using additional lineage or differentiation markers, such as Prospero and Pdm1.

We have now provided additional details regarding ISC differentiation upon NF-YA loss. As suggested by the reviewer, we have used antibodies against Prospero and Pdm1 to identify EE and EC cells, respectively. Our results show that loss of NF-YA in ISCs does not prevent progenitors from differentiating, even though the cells often retain the YFP signal of the ISC- Gal4>YFP. These results are in line with our esgReDDM analysis, which also shows that differentiated progeny are produced. We have now included this new data in Figure S1G, H.

[Minor points]
(i) The authors may also consider reflecting the nutrient-dependent aspect of their findings more explicitly in the title, as this represents a central component of the study.
We have now modified the title to cover the nutrient-dependent aspect of NF-Y in regulating intestinal growth. The new title is "Nuclear Factor Y controls nutrient-adaptive epithelial growth by regulating mTOR in the Drosophila midgut".

(ii) L125-126: The description of NF-Y as an "obligate inhibitor" appears too strong and should be softened.
We have revised the sentence from 'NF-Y is an obligate inhibitor' to ' NF-Y inhibits'.

(iii) L262: The fixation conditions used for immunostaining (8% PFA) are relatively strong and would benefit from a brief justification.
We have included a reference for the immunostaining protocol and an explanation of the strong fixation conditions used. Specifically, we routinely incubate midguts with primary antibodies for at least 48 h (rather than the recommended 24 h), which in most cases enhances the signal. Thus, stronger fixation is needed to preserve the tissue in optimal elasticity for mounting.

Second decision letter

MS ID#: dev.205643R1

MS TITLE: Nuclear Factor Y controls nutrient-adaptive epithelial growth by regulating mTOR in the Drosophila midgut

AUTHORS: Tetiana Strutynska, Onur Deniz and Jaakko Mattila

Dear Dr Mattila,

I am happy to tell you that your manuscript has been accepted for publication in Development, pending our standard publication integrity checks. It was accepted on 29 Jun 2026.

Reviewer 1

© 2026. Published by The Company of Biologists under the terms of the Creative Commons Attribution License (https://creativecommons.org/licenses/by/4.0/).

The study "Nuclear Factor Y controls epithelial growth by regulating mTOR in the Drosophila midgut" by Tetiana Strutynska, Onur Deniz, and Jaakko Mattila identifies the Drosophila NF-Y ortholog as a regulator of intestinal stem cell (ISC) proliferation and differentiation. The authors demonstrate that NF-Y acts as an essential inhibitor of mTORC1 signaling in ISCs, thereby preventing excessive proliferation, progenitor cell growth, and epithelial disruption under nutrient-rich conditions.

Overall, the manuscript provides compelling and converging lines of evidence supporting the proposed model. The experiments are well designed and convincing. The study is both interesting and relevant to a broad audience. The authors have adequately addressed most of my comments, and I believe that the manuscript now meets the requirements for publication.

Reviewer 2

SUMMARY OF THE ADVANCE MADE IN THIS PAPER AND ITS POTENTIAL SIGNIFICANCE TO THE FIELD

In this manuscript, Strutynska et al. investigate the role of the transcription factor NF-Y in regulating intestinal epithelial growth in the Drosophila midgut. The authors present evidence that NF-Y restricts intestinal stem cell (ISC) proliferation, progenitor growth, and epithelial expansion, and propose that this occurs through modulation of mTORC1 signaling. The study integrates genetic perturbations, lineage tracing, transcriptomic profiling, ultrastructural analysis, and pharmacological approaches to support a model in which NF-Y functions as a metabolic and proliferative brake.

Overall, this is a well-executed and clearly written study addressing an important question in tissue homeostasis. The integration of stem cell biology with metabolic regulation is a particular strength, and many of the experimental observations are convincing.

SUGGESTIONS TO AUTHORS

The reviewer is satisfied with the revisions made by the authors. The authors have adequately addressed the reviewers' concerns. The reviewer has no further comments on the manuscript.

© 2026. Published by The Company of Biologists under the terms of the Creative Commons Attribution License (https://creativecommons.org/licenses/by/4.0/).
